# A Case of Inversion of the Uterus

**Published:** 1861-08

**Authors:** James P. White

**Affiliations:** Prof. of Obstetrics in the University of Buffalo; Buffalo


ART. III.—*A Case of Inversion of the Uterus, by James P. White, M. D., Prof. of Obstetrics in the University of Buffalo.*

On Saturday, Dec. 8, 1860, Dr. T. T. Lockwood, (late Mayor of this city,) a highly respectable practitioner of more than twenty-five years' experience, called upon me to visit Mrs. R——, on Seventh street, with all possible despatch. Dr. L. informed me that Mrs. R. had previously been twice attended in labor by him, on which occasions nothing untoward or extraordinary had occurred. He had however, remarked that though a woman of small stature, her pelvis seemed to him remarkably large, and that she had completed the second stage of labor each time with great rapidity. He had been called to Mrs. R. this morning, she being not yet twenty-three years old, to attend her in her third labor. The first stage of labor, during which there were no unusual symptoms present, had occupied a little more than two hours, the patient not being at all exhausted, and was completed a little after 12 o'clock. At this time the membranes ruptured, a large amount of water was suddenly discharged, and with the next pain the child was thrown entirely into the world. Dr. L. immediately slipped the cord, which was of rather more than ordinary length, and once *around* the *child's neck,* but *not tightly drawn,* over its head. Soon after, as the child was crying vigorously, he tied and divided it, handing over the infant to the nurse.

His attention was immediately called to the mother, whom he now observed to look pale and to be fainting. Upon passing his hand up between her thighs, without having made the *cord tense,* he discovered a large tumour occupying the entire space, which, upon examination, he found to consist of the uterus, with the placenta attached. He immediately detached the placenta, carried the uterus up into the pelvis, entirely out of sight, where meeting with resistance to further reduction, he gave the woman a large draught of brandy and paregoric, and drove up to my house as rapidly as possible.

Without loss of time I returned with the Dr. to the residence of Mrs. R. whom we found surrounded by her friends, who supposed her dying. The woman was nearly pulseless, with all the symptoms of suffering from severe shock present in an eminent degree.

We immediately administered more brandy, tinc. opium and ammonia, and as she aroused a little and consciousness partially returned, we deemed it best to proceed at once with efforts to reposit the uterus. Upon introducing my hand into the vagina, I found the fundus of the uterus resting upon the perineum, though *entirely covered by the external organs.* Carrying the hand farther up, the inversion was found to be *complete,* and the organ pretty firmly contracted. The left hand placed upon the supra pubic region, detected a tumor there, which, although not round like the fundus of the uterus might, through abdominal pareities of moderate t hickness, not unlikely be mistaken for the normally contracted uterus in its natural position. Gentle continuous pressure was now made by the intra vaginal hand, whilst with the one upon the hyprogastrium the anterior lip was hooked or held by the fingers, and counter pressure exerted so as to prevent injury to the utero-vaginal connection. In a few minutes the neck began to be reflected upon the body, and although some dimpling or depression could be felt in the fundus, reduction took place from theneck to the body, and finally the fundus following the body up through the neck to its natural position. It should be remarked that, notwithstanding the relaxation of the system consequent upon syncope, and although not more than an hour or an hour and a quarter elapsed from the time of the delivery of the child to the complete *re-position* of the inverted uterus, the organic contraction was so firm as to require considerable pressure to carry the body through the os, and reflect the organ upon itself.

The patient was now laid in a comfortable position, with the head depressed and stimulants and opiates freely given. She soon began to revive, the pulse returned to the wrist, consciousness was restored, and at 3 P. M. about two hours after the operation, I left her with Dr. Lockwood. The amount of blood lost was not great, and was, in my opinion, utterly insufficient to account for the depression which immediately succeeded inversion, and continued for some hours after restoration. Indeed it is not unlikely that the patient would have lost her life from shock had not stimulants been freely and repeatedly given. The vital energies were so much depressed—the pulse not to be felt at the wrist—and the countenance was so ghastly that we did not venture to make the least effort at restoration of the inverted organ until she had taken stimulants freely. And yet there had been very little blood lost—for several days Mrs. R. remained very feeble, with frequent pulse, and tender hypogastrium, but gradually convalesced. Her restoration has been complete, and she is now in good health, nursing her infant and attending to her ordinary domestic duties.

*Remarks.*—The subject of inversio-uteri has only within the last few years been brought prominently before the profession, and many questions in relation to its occurrence and restoration still remain unsettled. Some of these points the case above reported may aid us in solving. In a medico-legal investigation of great interest, recently had in Chicago, it was maintained by counsel and this position was also sustained by medical testimony, that, it was impossible for the recently inverted uterus occurring immediately after delivery, at the full period of gestation *to remain in the vagina.* It was by those who sustained this theory held that the long diameter of the uterus would be such as to render it necessary that it should protrude at the vulva. In previous efforts to reduce the recently inverted organ I was certain that the fundus was carried completely within the vulva, before reduction at the neck commenced, and therefore stated my belief in its possibility. The vagina which has been elongated by pregnancy, and finally dilated by the passage of a fully developed fœtal head, may be carried up into the abdominal cavity quite above the summit of the pubis, and thus increase its longitudinal capacity as well as its diameter. Speculate as we may, here was a patient delivered of a full grown male child, the inverted uterus following immediately, and within a very short space of time carried back completely into the vagina, and there left for more than half an hour without any pressure or support being made at the perineum. During this period the head of the patient was repeatedly elevated, to take stimulants and opiates, and she made considerable effort in throwing herself about in approaching syncope. During the time the uterus remained there Dr. Lockwood left the house and came himself for me, in order to secure my early attendance, and yet some minutes after my arrival, upon examination, I found the fundus *did not* project beyond the labia. My attention having been recently called to this point, I was especially careful to note its position. More than this, when pressure was applied to the fundus for the purpose of re-position, the fundus was carried still higher before the os commenced to be reflected over the neck. In this as in former recent cases reported by me, I found great assistance in the effort at restoration by "hooking" the fingers of the left hand over the anterior uterine lip, which could be distinctly felt through abdominal parietes of moderate thickness. It can be readily conceived by any one who has felt the tumor formed by the uterine os and neck in the hypogastrium in inversion, that it might be mistaken for the fundus, and the inexperienced practitioner be led to suppose it was the fundus, and that the organ occupied its natural relations.

Again, much discussion has been had relative to the manner in which reposition is effected. In this, as in all the preceding cases, I was careful to note that the os was first doubled down upon the neck, and then upon the body, and finally over the fundus. It is true that pressure upon the fundus produced slight depression or dimpling at its summit, but this did not increase and go on to complete re-inversion. In the seven cases of inversion, recent as well as chronic, in which I have reposited the uterus at various dates, varying from a few minutes to more than fifteen years after its occurrence, in all, so far as I am able to judge, restoration has been accomplished in the manner above described, and in no instance by pushing the fundus up through the body and neck.—Again, the manner of its occurrence in this case deserves to be especially noted. It would seem to have been *spontaneous*, as the funis was not tense, and was of more than the ordinary length. Dr. Lockwood, who is a highly respectable accoucher, of large experience, assures me that when he slipped the cord over the head of the child, it was quite loose, and required no tractile effort to accomplish it. He assures me also that no traction was made upon the cord after the birth of the child, before the sinking condition of the patient claimed his attention, when, upon examination, he found the uterine tumor between her thighs with the placenta attached. It would seem that the uterus, which was excited to energetic contractions by the difficulty of dilating the os, meeting with little resistance in pushing its contents through the pelvis, followed by its own contraction at the fundus, the suddenly rejected ovum down into and through the neck, and os, and into the world. I have never been present when the accident occurred, but it is not unlikely that it may occur immediately after sudden delivery, *spontaneously*, although doubtless much more frequently to be charged to unskillful or rude manipulations. This is the second case which has now come under my observation in which the inversion could not be attributed to forcible pulling upon the cord or placenta by the accoucher in attendance. It would not be safe to infer that in the remaining five cases traction at the cord or placena, was the sole cause, as their history could not be very accurately established. Two which are well authenticated, are sufficient, however, to establish the fact that, it *may* occur in the hands of good practitioners, when the second stage is rapidly completed, unless great caution be taken to retard the sudden expulsion of the ovum. Spontaneous inversion of the organ can, however, only take place immediately after the expulsion of the fœtus, and not, as has been erroneously maintained, at a period remote from delivery, and after tonic contraction of the organ has been established.

BUFFALO, July 12, 1861.

